# The *Streptococcus pneumoniae* Pilus-1 Displays a Biphasic Expression Pattern

**DOI:** 10.1371/journal.pone.0021269

**Published:** 2011-06-22

**Authors:** Gabriella De Angelis, Monica Moschioni, Alessandro Muzzi, Alfredo Pezzicoli, Stefano Censini, Isabel Delany, Morena Lo Sapio, Antonia Sinisi, Claudio Donati, Vega Masignani, Michèle A. Barocchi

**Affiliations:** Novartis Vaccines and Diagnostics, Siena, Italy; Instituto Butantan, Brazil

## Abstract

The *Streptococcus pneumoniae* pilus-1 is encoded by pilus islet 1 (PI-1), which has three clonal variants (clade I, II and III) and is present in about 30% of clinical pneumococcal isolates. *In vitro* and *in vivo* assays have demonstrated that pilus-1 is involved in attachment to epithelial cells and virulence, as well as protection in mouse models of infection. Several reports suggest that pilus-1 expression is tightly regulated and involves the interplay of numerous genetic regulators, including the PI-1 positive regulator RlrA. In this report we provide evidence that pilus expression, when analyzed at the single-cell level in PI-1 positive strains, is biphasic. In fact, the strains present two phenotypically different sub-populations of bacteria, one that expresses the pilus, while the other does not. The proportions of these two phenotypes are variable among the strains tested and are not influenced by genotype, serotype, growth conditions, colony morphology or by the presence of antibodies directed toward the pilus components. Two sub-populations, enriched in pilus expressing or not expressing bacteria were obtained by means of colony selection and immuno-detection methods for five strains. PI-1 sequencing in the two sub-populations revealed the absence of mutations, thus indicating that the biphasic expression observed is not due to a genetic modification within PI-1. Microarray expression profile and western blot analyses on whole bacterial lysates performed comparing the two enriched sub-populations, revealed that pilus expression is regulated at the transcriptional level (on/off regulation), and that there are no other genes, in addition to those encoded by PI-1, concurrently regulated across the strains tested. Finally, we provide evidence that the over-expression of the RrlA positive regulator is sufficient to induce pilus expression in pilus-1 negative bacteria. Overall, the data presented here suggest that the observed biphasic pilus expression phenotype could be an example of bistability in pneumococcus.

## Introduction

*Streptococcus pneumoniae* (*S. pneumoniae*) is a commensal pathogen associated with severe diseases such as meningitis and bacteraemia as well as pneumonia, sinusitis and otitis media [1]–[6]. In fact, despite its pathogenic potential, the pneumococcus is a common component of the human nasopharyngeal tract of children and healthy adults [7]. This carriage state can persist for several months and can be regarded as a risk factor for the development of respiratory diseases, and also as the source for pneumococcal transmission to other individuals [8]–[10]. Several pneumococcal virulence factors have been identified, however, little is known about the fundamental disease determinants and how they are expressed during the development of disease [11]–[13]. *S. pneumoniae*, like most streptococci, decorates its surface with long multimeric filaments known as pili composed of covalently linked subunits [14]–[17]. Although their biological function has not been fully elucidated, pneumococcal pili have been associated with virulence and the capability of the microorganism to better adhere to epithelial cells and to colonize the nasopharynx [18], [19]. In addition, pilus-1 subunits have been shown to confer protection in invasive mouse models of infection, and are therefore regarded as potential candidates for a new generation of protein-based vaccines targeting *S. pneumoniae* diseases [20], [21]. The pneumococcal pilus is encoded by the pilus islet 1 (PI-1), a 12 kb locus, containing seven genes encoding a transcriptional regulator (RlrA), which positively regulates pilus expression [22] and its own expression, three pilus structural subunits (RrgA, RrgB and RrgC) and three sortase enzymes (SrtC-1, SrtC-2 and SrtC-3), which covalently assemble the pilus subunits on the bacterial surface [23]–[26]. Several molecular epidemiological reports highlight that PI-1 is present in about 30% of the pneumococcal isolates, regardless of the geographical origin and the disease outcome analyzed [17], [27]–[29]. PI-1 is clonally inherited by *S. pneumoniae* strains, and its presence is associated with the genotype of the isolates rather than the serotype. PI-1 exists in three variants, namely clade I, II and III. Since each variant is associated with specific clones, PI-1 clades display different regional prevalence, strictly depending on the distribution of the clones [17], [28]. Most of the PI-1 variability is concentrated in the genes coding for the pilus components: RrgB, the main pilus subunit, and RrgA, which is the major adhesin.

Given the potentially serious implications that the pilus might have for disease and transmission, several reports have focused on the evaluation of genetic regulators that are able to modulate pilus expression and therefore bacterial virulence. Seven proteins, in addition to the PI-1 positive regulator RlrA, were demonstrated by different groups to negatively influence *in vitro* pilus expression levels (MgrA, HK343, MerR, CbpS, TCS08, mntE, PsaR) [30]–[33].

However, it is still not clear whether all PI-1 positive pneumococci express pili (*in vitro* and *in vivo*), and if genetic differences and growth conditions can influence pilus-1 expression levels. In this work, we analyze pilus-1 expression in a panel of 139 *S. pneumoniae* clinical strains, and provide evidence that all of the strains tested, regardless of serotype, genotype and growth conditions, present two phenotypically distinct sub-populations, expressing the pilus at high (here defined as Pil+) and undetectable levels (here defined as Pil-). In addition, the proportion of Pil+/Pil- was not influenced by the presence of anti-RrgB antibodies during the growth. To better elucidate the pilus expression phenotype, for a number of strains we separated to about 95% purity two bacterial sub-populations, enriched either in pilus expressing or pilus non-expressing pneumococci. PI-1 sequence analysis of the two sub-populations reveals the absence of genetic phase variation events within the islet. Finally, we compare the expression profiles of the two sub-populations in a panel of *S. pneumoniae* clinical strains and conclude that: 1) pilus expression is regulated at the transcriptional level, 2) this regulation involves all PI-1 components, and 3) there are no other genes in addition to those encoded by PI-1 concurrently regulated across the isolates tested.

## Results

### Pilus-1 has a biphasic expression pattern

**Figure 1:**
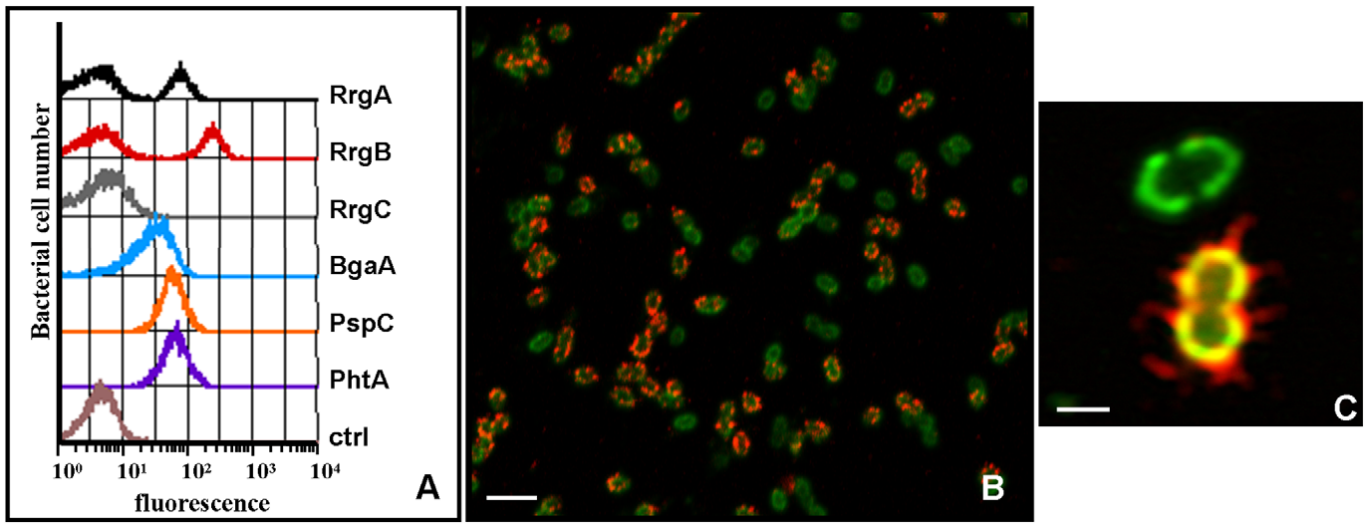
Pilus components display a biphasic expression pattern. A) TIGR4 bacteria were labeled with anti-RrgA, RrgB clade I, RrgC, BgaA (beta-galactosidase), PhtA (pneumococcal histidine triad protein A) or PspC (pneumococcal surface protein C, also known as CbpA,) primary antibodies (1∶400 dilution), and with FITC anti-mouse IgG secondary antibodies (1∶100 dilution). Bacterial staining was analyzed by flow cytometry (FACS-Calibur). Sera of mice immunized with PBS were used as negative control. B,C) TIGR4 bacteria were processed for immunofluorescence, stained with mouse anti-RrgB antibodies (1∶2000 dilution) (red) and with *S. pneumoniae* anti-capsular antibodies (Omniserum 1∶2000 dilution) (green). Imaging was performed with a confocal microscope. Scale bar is 4 µm in panel B and 1 µm in panel C.

In order to elucidate pilus expression in *S. pneumoniae*, TIGR4 bacteria were grown, stained with antibodies raised against the pilus components (RrgA, RrgB, and RrgC) and three surface exposed proteins (PspC, PhtA and BgaA), and then analyzed by flow cytometry (Fig. 1A) [34]–[37]. While the bacteria were found to uniformly express PspC, PhtA and BgaA, the specific antibodies for RrgA and RrgB revealed the presence of two sub-populations, one expressing high levels of the pilus subunits (Pil+), while the other displayed a mean fluorescence intensity (MFI) comparable to the negative control (sera raised against an unrelated protein) (Pil-). Interestingly, the analysis of bacteria labeled with anti-RrgC antibodies revealed a single homogeneous population with an MFI similar to the negative control, confirming that, in intact bacteria, RrgC is not exposed on the bacterial surface [15]. To support the data obtained by flow cytometry, the bacteria were incubated with the antisera and then analyzed by immunofluorescence. As shown in Figure 1B (and in the enlargement of Fig. 1C), bacteria were uniformly stained by anti-capsular antibodies, whereas the RrgB pilus specific signal was present only in a subset of bacteria (Pil+) and undetectable in the others (Pil−).

### *S. pneumoniae* pilus expression is not correlated with genotype, clade type and serotype

**Figure 2:**
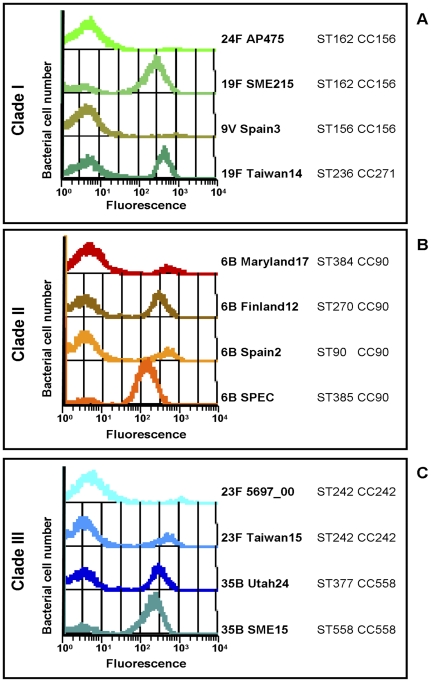
Pilus expression ratio is not correlated with serotype, genotype or clade type. Bacteria containing either PI-1 clade I (A), clade II (B) or clade III (C), were labeled with clade specific anti-RrgB antibodies (1∶400 dilution) and FITC anti-mouse IgG secondary antibodies (1∶100 dilution). Pilus-1 expression was then analyzed by flow cytometry (FACS-Calibur).

Given the biphasic expression pattern observed in the TIGR4 strain, a collection of 139 strains was selected out of 436 *S. pneumoniae* PI-1 positive strains (*see* materials and methods) and analyzed for pilus-1 expression by FACS analysis. All of the selected strains revealed a biphasic pilus expression, with the proportion of Pil+ bacteria ranging from 5 to 95%. As presented in figure 2 for a selection of strains, there was no correlation between the ratio of Pil+ versus Pil- bacteria (pilus expression ratio) and the genotype, clade type and serotype. In addition, there was no association with the disease outcome of the isolates, as the pilus expression ratio was heterogeneous in invasive, carriage and otitis media strains from the same or different geographical origins (data not shown).

A detailed analysis of the PI-1 sequence revealed the presence of variable short nucleotide repeats in the intergenic regions upstream RrgA (2-6 CTATA repeats) and RrgB (poly-A tract containing 5 or 6 adenosine nucleotides). Since in other organisms the presence of different repeat numbers in the promoter region accounts for variable expression of the downstream gene [38]–[40], we hypothesized that these sequence repeats could act as regulation signals for RrgA and RrgB-RrgC expression. However, we found no correlation between the number of repeats and the pilus expression ratio in the 44 strains analyzed [41] (data not shown).

### Pilus expression ratio remains unchanged growing the bacteria under different conditions or in the presence of RrgB antisera

The identification of factors able to modulate pilus-1 expression *in vitro*, and, in particular, to enhance the pilus expression ratio could facilitate the understanding of the pilus role *in vivo*. Therefore, three *S. pneumoniae* strains (TIGR4, 6B Finland 12 and 35B SME 15) were grown under several growth conditions (for details *see* materials and methods) and pilus-1 expression evaluated by flow cytometry, revealing that pilus-1 expression remained unchanged (Fig. S1). Only the addition of 5-10% fresh sheep blood to CDM medium slightly increased the pilus-1 expression ratio to variable extents in TIGR4, but the increase was not reproducible in the other two strains (data not shown). Since the pilus components are potential protein-based vaccine candidates, it is crucial to understand if the pilus expression ratio is influenced by the presence of antibodies directed toward pilus components. The three *S. pneumoniae* strains were therefore grown in the presence of antibodies directed against RrgB (sera raised against BgaA were used as negative control), and were allowed to replicate seven cycles. The expression of pilus-1 was then verified by FACS analysis revealing that the pilus expression ratio was unaffected by the presence of the RrgB antisera (data not shown).

### Two *S. pneumoniae* sub-populations enriched in Pil+ or Pil- bacteria can be separated by colony selection

**Figure 3:**
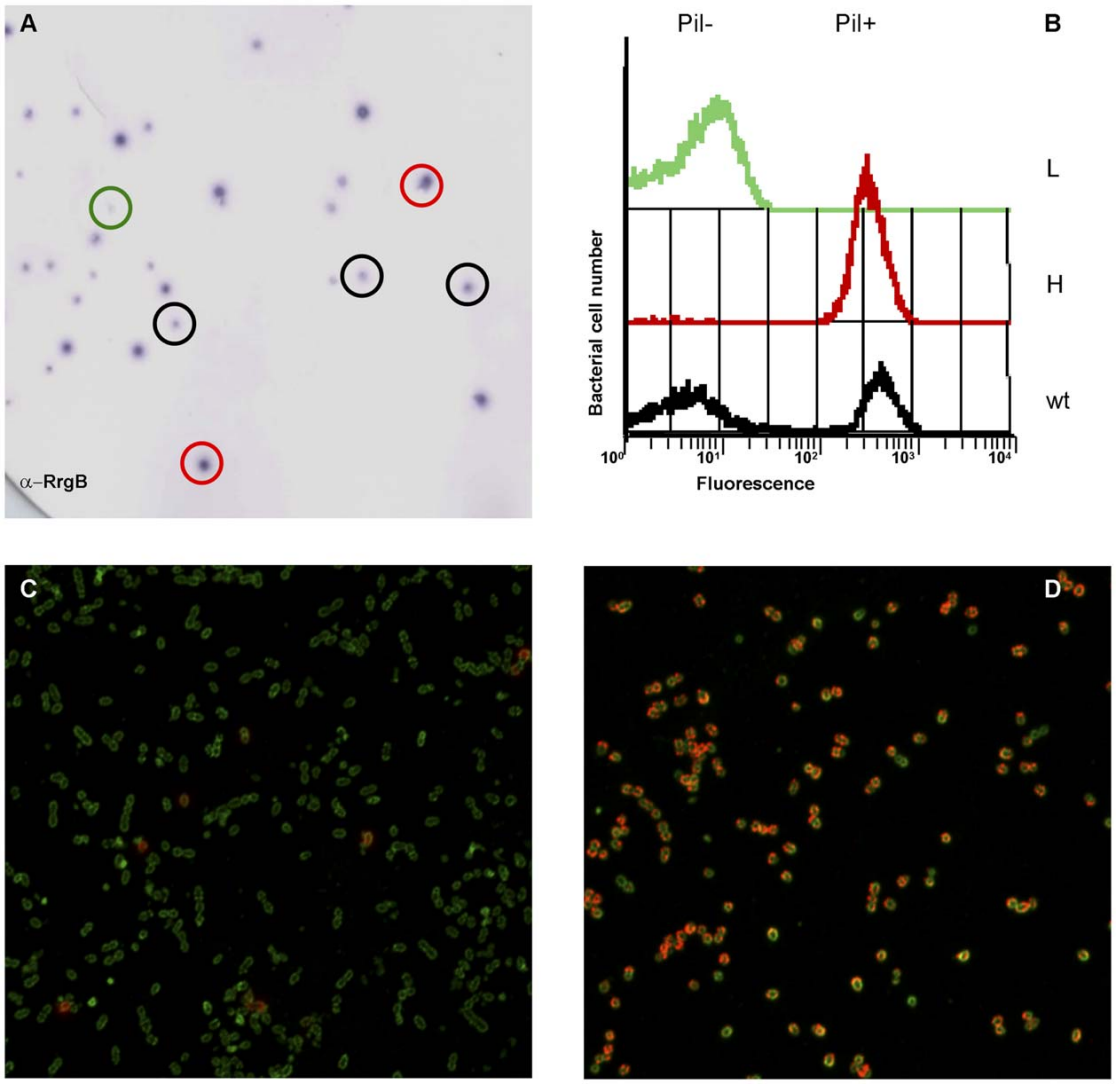
Stable separation of enriched high (H) and low (L) pilus-1 expressing sub-populations. A) TIGR4 pilus-1 expression was revealed on single colonies by colony immunoblot using anti-RrgB clade I antibodies (green, black and red circles correspond to colonies displaying low, medium or high RrgB specific signal intensities, respectively). Bacteria recovered from the growth of different colonies were stained with anti-RrgB clade I antibodies and analyzed by flow cytometry (B). The bacteria expressing (Pil+) and non- expressing (Pil-) the pilus-1 are indicated in the L (green) and H (red) enriched sub-populations, and in the wt (black). H and L sub-populations were stained for immunofluorescence (C and D). Bacteria were incubated with mouse anti-RrgB antibodies (1∶2000 dilution) (red) and with *S.pneumoniae* anti-capsular antibodies (Omniserum 1∶2000 dilution) (green). Imaging was performed with a confocal microscope. Scale bar is 5 µm.

In order to verify if Pil+ and Pil- bacteria after duplication maintain their original pilus expression phenotype, bacteria, following a sonication step, were grown on a plate as single colonies and then analyzed for pilus expression. As shown in figure 3A, when colony blot was performed with anti-RrgB antibodies, the colonies displayed different RrgB intensities, but none were RrgB negative.

Colonies showing differential RrgB staining were selected and re-grown. Analysis performed by flow cytometry revealed that the majority of the colonies gave rise to populations with a ratio of pilus expression similar to the original strain. However, some colonies gave rise to either mostly Pil+ or Pil- sub-populations, defined as H (high pilus expression) or L (low pilus expression) sub-populations (Fig. 3B). Despite numerous attempts, completely positive or negative sub-populations were never obtained, as there were always Pil+ and Pil- in the L and H sub-populations, respectively (Fig. 3C and 3D). Notably, the two enriched sub-populations (H and L) were stably maintained after consecutive re-growths and long-term storage at -80°C.

In addition, since in *S. pneumoniae* phase variability is commonly associated with the widely known colony morphology phenotype variation (opaque/transparent) [42]–[45], we sought to determine if different colony opacity was related to a specific pilus expression ratio. Bacteria grown on a plate as single colonies, following the identification of different morphology (opaque or transparent), were processed by immuno-blot analysis with anti RrgB antibodies. Interestingly, the different RrgB intensities observed (corresponding to different pilus expression ratios) were completely unrelated to the colony morphology.

### PI-1 components expression is undetectable in pilus-1 negative bacteria

**Figure 4:**
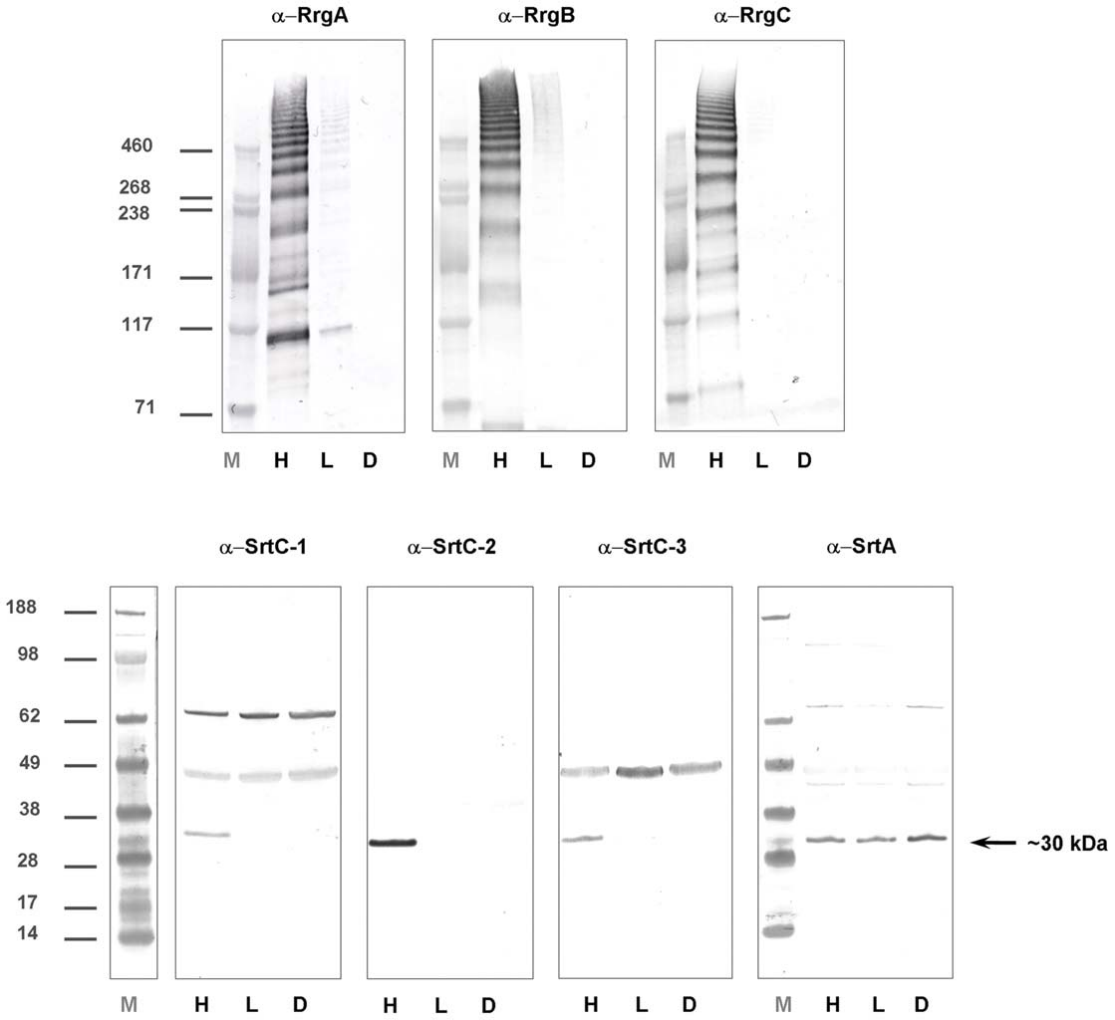
PI-1 encoded proteins are not expressed in RrgB negative bacteria. WB analysis performed on whole bacterial lysates of TIGR4 H (H), L (L) or TIGR4 L depleted of RrgB positive bacteria (D), using polyclonal mouse antisera against RrgA, RrgB, RrgC (see High molecular weight ladders), SrtC-1, SrtC-2 ,SrtC-3 (see bands indicated by arrows) and SrtA (used as loading control).

The *S. pneumoniae* pilus-1 polymerization is a complex and tightly coordinated process, not yet fully elucidated, requiring the simultaneous involvement of pilus components and bacterial sortases [23], [46], [47]. Following this observation, and in order to gain more insight into the pilus polymerization mechanism, the expression levels of the single PI-1 components were evaluated in Pil- bacteria. In this regard, whole bacterial lysates of the TIGR4H and TIGR4L sub-populations, along with a TIGR4L sub-population further depleted of pilus positive bacteria (TIGR4 D)(*see* materials and methods) were probed with antibodies raised against RrgA, B and C and SrtC1, 2 and 3 and SrtA (used as experimental control). As reported in figure 4, the impossibility to detect RrgA and RrgB on the surface correlates with the lack of expression of all PI-1 components, both pilus-1 subunits and PI-1 sortases (as demonstrated by the absence of both the typical pilus HMW ladder and protein monomers in TIGR4D).

### Biphasic pilus expression is not due to phase variation within the PI-1

Population heterogeneity of protein expression can result from either genetic rearrangement, as is the case in phase variation, or DNA modification, such as methylation, or it can be due to epigenetic phenotypic variation (not caused by a change in DNA sequence). In particular, phase variation is governed by random frameshift mutations or site-specific recombination events, occurring in open reading frames or promoter regions, and resulting in a variable expression of a specific protein [48], [49]. To exclude that the biphasic pilus expression pattern was due to phase variation events at the level of the positive regulator (*rlrA*), or, more in general, to point mutations within PI-1, the islet was sequenced for three strains (TIGR4, 19FTaiwan14, OREP4) in the two enriched sub-populations (L and H) and in the wild-type. Neither alterations in the genomic sequence nor uncertainties in the chromatograms were observed, indicating that intra-islet gene mutations or recombination events within PI-1 are not responsible for the pilus-1 expression pattern.

### Only PI-1 components are differentially regulated between the H and L pilus expressing sub-populations

**Figure 5:**
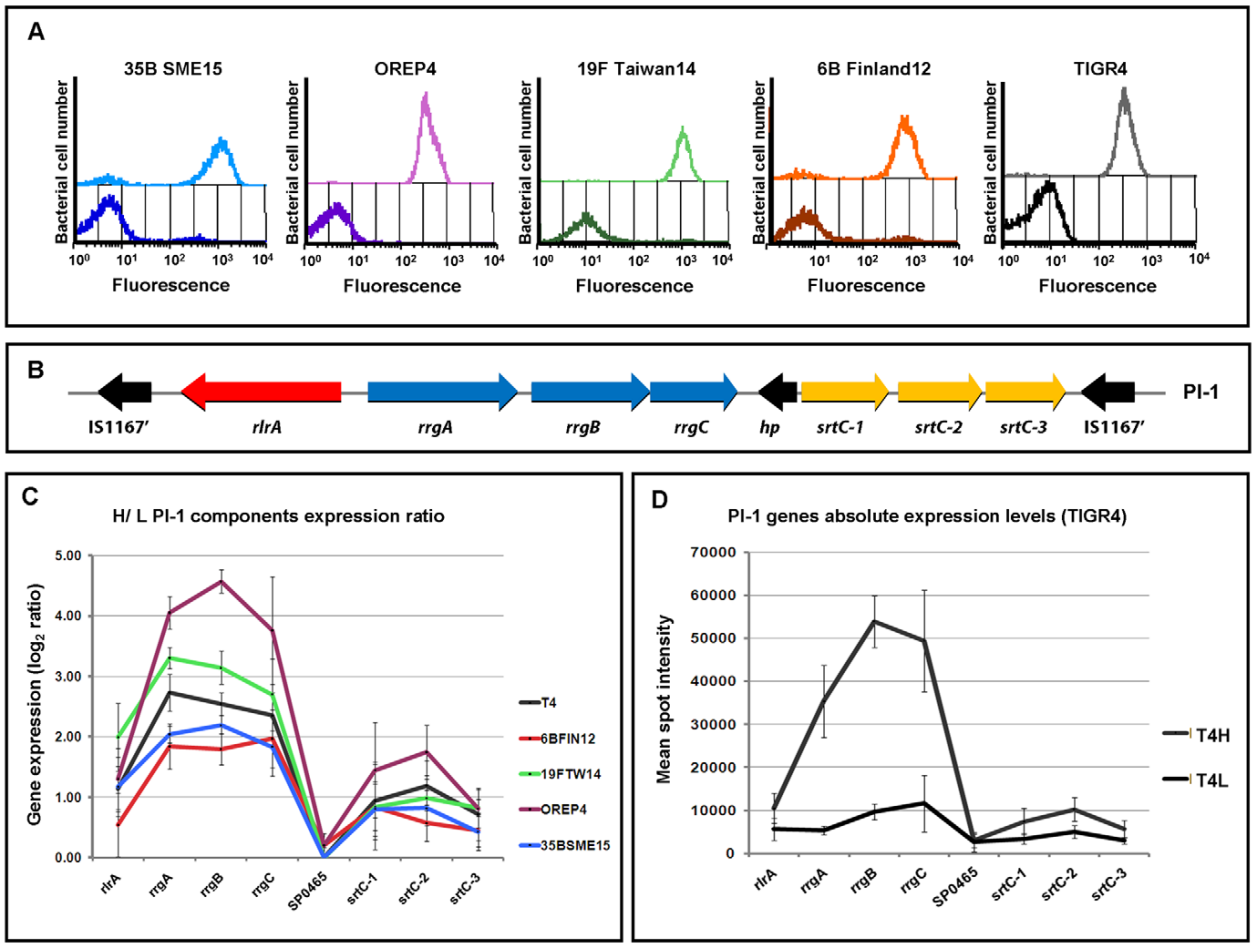
Pilus-1 expression is regulated at the transcriptional level. **A)** High and low pilus expressing sub-populations of strains TIGR4 (Clade I), 19F Taiwan 14 (Clade I), OREP4 (Clade I), 6B Finland 14 (Clade II) and 35B SME 15 (Clade III) were labeled with clade specific anti-RrgB antibodies (1∶400 dilution) and FITC anti-mouse IgG secondary antibodies (1∶100 dilution). Pilus-1 expression was then analyzed by flow cytometry (FACS-Calibur). **B)** Schematic representation of PI-1. **C)** Log_2_ ratio values indicating the PI-1 genes differential expression in High vs. Low pilus expressing sub-populations in the five above mentioned strains, as measured by spotted DNA microarray analysis. The data are measures of relative gene expression during *in vitro* growth in liquid cultures. The values reported for each gene are the mean of all the spots and their replicates within the array and of two independent experiments (bars represent standard deviations). **D)** Absolute gene expression levels of PI-1 genes measured for TIGR4 high and low pilus expressing sub-populations by microarray hybridization. Absolute expression levels reported for each gene are the mean of all the spots and their replicates within the array and of two independent experiments (bars represent the obtained standard deviations).

To evaluate if pilus-1 expression was regulated at the transcriptional or translational level, total RNAs were extracted from the H and L sub-populations of five strains, TIGR4, 19F Taiwan 14, OREP4 (Clade I), 6BFin12 (Clade II) and 35B SME 15 (Clade III) (Fig. 5A). The expression profiles of the L versus the H pilus expressing sub-populations were directly compared by microarray analysis (*see* materials and methods). As shown in figure 5C, the analysis of the log_2_ H/L signal intensity ratio curves for PI-1 components in the five strains revealed that all the PI-1 genes (including *rlrA*) were differentially regulated in all the strains tested. The different log_2_ signal intensity ratios observed among the isolates clearly depends on the ability to enrich in Pil+ and Pil- the H and L sub-populations, respectively. In fact, the strains 6B Finland 12 and 35B SME 15 showing the lowest log_2_ H/L signal intensity ratio, were the isolates with the least enriched sub-populations (Fig. 5A). In addition, the log_2_ ratio expression levels measured for the three sortases were consistently lower with respect to the pilus components and the *rlrA* regulator (Fig. 5C). This result is dependent on the different absolute expression levels measured for the PI-1 genes both within the H and the L pilus expressing sub-populations (sortase absolute expression levels were about four-five times lower than pilus components) as reported in figure 5D for the TIGR4 strain. This observation suggests the presence of multiple promoters within PI-1, upstream *rlrA*, *rrgA*, *rrgB* and *srtC-1* as previously published [22]. In addition, the data obtained further confirm the absence of a mRNA coding for the protein annotated as hypothetical protein SP0465 [50]. The ratios of H/L obtained for the PI-1 genes with the microarray transcriptome analysis were further confirmed by qRT-PCR (*see* materials and methods).

Interestingly, within the resolution limits of the microarray analysis, no other conserved genes apart from PI-1 components were identified as concurrently regulated in all the five strains tested when we extended the expression profile analysis of the L versus H pilus sub-populations to the complete genome (Fig. S2A, S2B). Remarkably, changes in the expression rate of previously published negative regulators [30]–[33] (expected to be up-regulated in the L pilus expressing sub-population) remained undetected by our assay (Fig. S2C).

### Expression of the RlrA regulator in Pil- bacteria is sufficient to induce pilus polymerization

**Figure 6:**
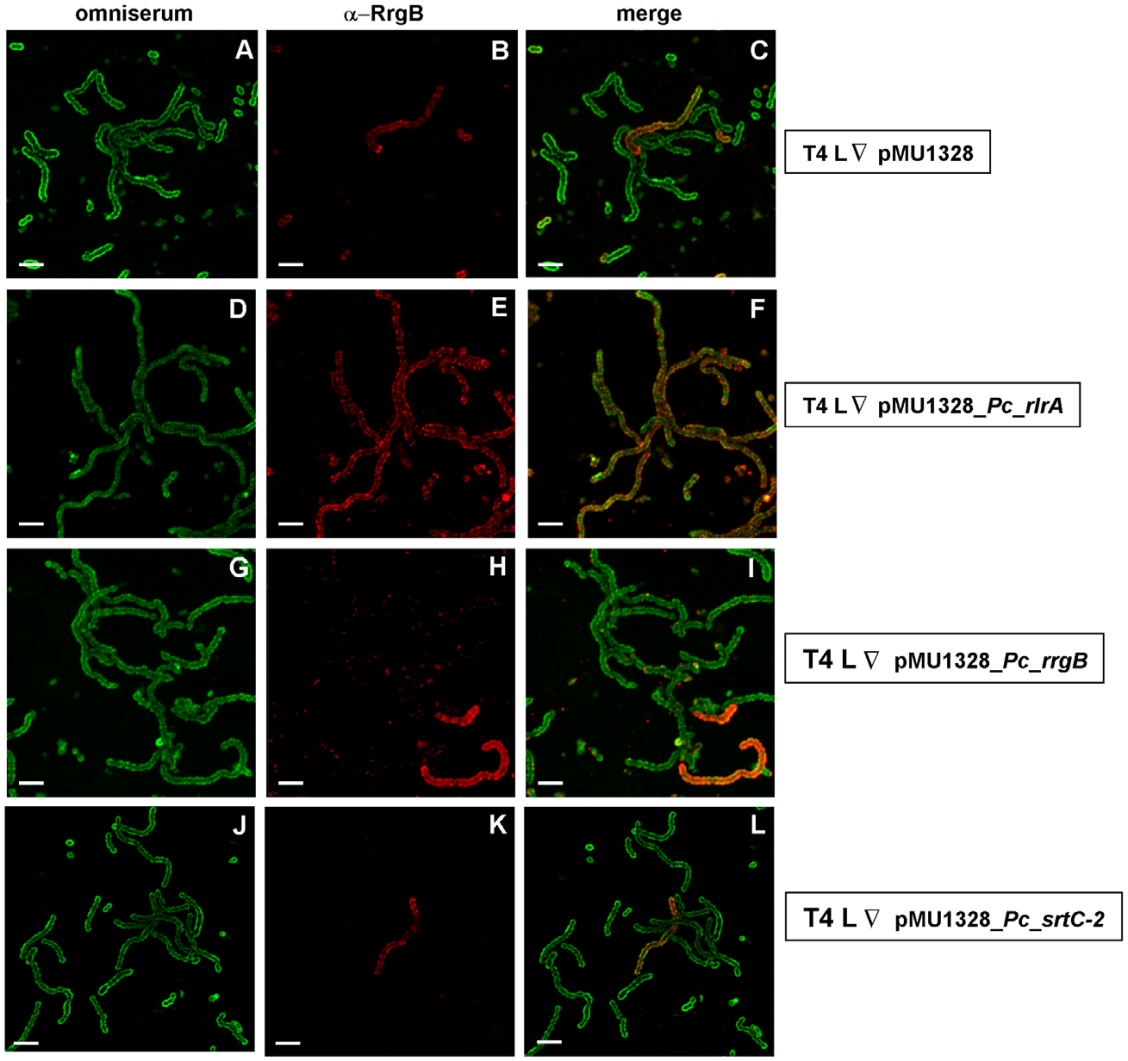
RlrA expression in pilus negative (Pil-) bacteria induces pilus polymerization. TIGR4 low pilus expressing bacteria transformed with *pMU1328* (panels A-C), *pMU1328-Pc-rlrA* (panels D-F), with *pMU1328-Pc-rrgB* (panels G-I) or with *pMU1328-Pc-srtC-2* (panels J-L) were processed for confocal microscopy immuno-fluorescence analysis by incubating *S. pneumoniae* with anti-capsular antibodies (Omniserum 1∶2000 dilution) (green, left panels) and mouse anti-RrgB antibodies (1∶2000 dilution) (red, central panels). Right panels represent the merged signal of the left and central panels. Scale bar is 4 µm.

In order to better evaluate the regulation of the pilus locus, the effects on the pilus polymerization induced by the expression within Pil- bacteria of RlrA (the positive regulator), RrgB or SrtC-2 were evaluated. Briefly, the TIGR4 L sub-population was transformed with a pMU1328 plasmid [51] containing the *rrgB,* the *srtC-2* or the *rlrA* gene (pMU1328 empty vector was used as negative control). Following transformation, bacteria were studied by FACS analysis, western blot (Fig. S3) and immunofluorescence (Fig. 6). The ratio of bacteria expressing the pilus was not altered upon transformation with the pMU1328 empty plasmid (Fig. 6 panels A-C), while the expression of RlrA induced polymerization of the pilus in 100% of the *S. pneumoniae* population (Fig. 6 panels D-F). Interestingly, following the expression of RrgB or SrtC-2 (Fig. 6 panels G-I and J-L, respectively) the proportion of bacteria able to polymerize the pilus on their surface did not change when compared to the control. In detail, when RrgB was over-expressed, RrgB was localized in clusters on the Pil- bacterial surface (Fig. 6 panels G-I), but remained un-polymerized (Fig. S3) due to the lack of expression of the pilus specific sortases. On the other hand, the expression of a functional SrtC-2 (Fig. 6 panels J-L and Fig. S3), which in the presence of the RrgB monomer is sufficient to induce RrgB polymerization (Fig. S3 and Text S1), did not induce in the T4L sub-population any change in pilus components expression or pilus polymerization. Taken together, these data indicate that the expression of RlrA, unlike that of RrgB and SrtC-2, was sufficient to induce the expression of all the other PI-1 components.

## Discussion

Following their first identification in other gram-positive bacteria, pili have been detected on the surface of the major human pathogen *S. pneumonia*e and shown to be immunogenic and involved in pathogenicity in *in vivo* and *in vitro* studies [18]–[20]. Epidemiological reports have defined that PI-1, coding for the proteins implicated in pilus-1 biogenesis, is present in approximately 30% of the pneumococcal isolates and exists in three genetically related variants [17], [28], [29].

The molecular structure of pilus-1 and the mechanism of pilus assembly have been investigated, and a number of putative PI-1 genetic regulators have been described [15], [26], [30]–[33], [52], [53]. However, still very little is known about the regulation of pilus expression, the environmental conditions able to modulate it and the complex macromolecular machinery that regulates pili biogenesis. In addition, in all the above mentioned studies, *S. pneumoniae* pilus expression has always been evaluated not on a single cell basis, but as an average behavior of a large population.

In this work, by using detection methods able to discriminate single cells, we have compared the expression of the pilus to other known surface exposed virulence factors in the laboratory reference strain TIGR4. Unlike the other proteins tested, pilus-1 components were found to display a biphasic expression pattern. The two phenotypically distinct sub-populations, Pil+ and Pil-, are present in variable ratios in all the strains tested, similarly to what has been previously observed for the transparent and opaque variants in the intra-strain colony morphology phase variation [44], [45], [54]. The pilus expression ratio is inherited by daughter cells and is not influenced by bacterial genetic and epidemiological characteristics, *in vitro* growth conditions, or growth in the presence of pilus antisera. In addition, in contrast to what reported for the opacity phenotype, where the opaque variant is the most frequent phenotype found among invasive and acute otitis media isolates and the transparent more associated to carriage [54], [55], the pilus expression ratios were similar in carriage and invasive isolates. Furthermore, the majority of the colonies isolated on solid medium from the same strain show a similar pilus expression pattern, thus indicating that this may be influenced by some genetic traits of the strain, still unidentified. However, some colonies generate bacterial populations displaying different ratios of Pil+/Pil- bacteria. For this reason, since the isolation of the clinical isolates from the human host always implies a process of *in vitro* growth and stochastic colony selection, the pilus expression ratios observed may not be representative of the expression of pili *in vivo.* This aspect needs further investigation.

Interestingly, despite numerous attempts using single colony selection, sub-populations containing 100% of either Pil+ or Pil− bacteria were never obtained. Consequently, the analysis of pilus expression (both by microarray and western blot) was performed by comparing two sub-populations, H and L, enriched in Pil+ and Pil− bacteria, respectively. Microarray expression profiling was only able to detect a significant change in expression for the PI-1 components, including the *rlrA* positive regulator, while changes in the expression of genes outside of the PI-1 in the five strains tested (H vs. L sub-populations) remained undetected. Moreover, as clearly demonstrated in this work, RlrA (unlike RrgB and SrtC-2) expression was sufficient to induce the polymerization of a functional pilus on Pil- pneumococci, resulting in the switch of pilus biosynthesis from an “off” to an “on” state. The latter observation is in agreement with previous reports identifying RlrA as the positive regulator of PI-1 genes transcription, able to activate its own transcription and to establish a positive feedback loop [22]. Additionally, this phenomenon is supported by the difference in the *rlrA* transcript observed in the H vs L sub-populations. Indeed, the *rlrA* transcriptional change is in the range of that observed for the three PI-1 sortases (and much lower than that observed for the structural subunits), thus in agreement with the idea that for regulatory proteins and enzymes (exerting catalytic activity), small transcriptional changes could be sufficient to induce an appreciable phenotypic difference. Taken together these data suggest two possible scenarios: i) pilus expression is modulated by the interplay of numerous regulators, but transcriptional changes outside PI-1 remained undetected by our assay as would be expected since very slight changes in a regulatory transcript could be responsible for important phenotypic changes, especially when elaborated like in this case indirectly through another regulator; ii) pilus expression is not dependent on regulators located outside PI-1, but direct changes are in response to unknown external stimuli or noise increasing the transcription of *rlrA* and thus switching “on” the pilus biosynthesis. Additional data obtained analyzing pilus expression at the single cell level in knock-out mutants of the known PI-1 repressors, seem to exclude the possibility of their direct involvement in pilus regulation (data not shown), and therefore favor the second hypothesis.

Although the molecular mechanisms triggering such regulation events are still not clear, the data presented in this work suggest that *S. pneumoniae* pilus expression could be an example of bistability, as it was recently suggested for *Streptococcus pyogenes* FCT3 encoded pili [56]. In fact, this term is usually referred to phenotypic variation examples where: 1) two stable expression states coexist within a population; 2) noise or different factors operate across the entire population driving cells to switch into the alternative expression state and determine the overall switching probability; 3) the regulation occurs through the presence of feedback loops, either positive or double negative [57]–[59]. Moreover, bistability is epigenetic in nature (not caused by a change in the DNA sequence). In this regard, our data exclude the possibility of phase variation events within PI-1, but genetic modifications present elsewhere in the genome (still unexplored) could indirectly influence pilus expression.

The molecular basis and the biological benefit of this bistability phenomenon are currently unknown. Presumably, such switching mechanisms have evolved as a way for bacteria to be phenotypically pre-adapted to survive present or pending adverse conditions. Most likely, this heterogeneity helps the bacterium to utilize different niches within an ecosystem, and even has the potential to increase the overall fitness of the species, and prepare a sub-population of *S. pneumoniae* cells to promptly adapt in response to stress or different environmental conditions.

In conclusion, this study has shown that pilus expression follows a biphasic pattern, and is an on-off regulated mechanism occurring at the transcriptional level and involving all the PI-1 components, included the PI-1 positive regulator RlrA (there could be multiple promoter regions within PI-1 responding to RlrA positive regulation). Further studies are necessary to better clarify the molecular mechanisms responsible for the biphasic pilus phenotype. This finding suggests that new experimental approaches should be devised to assess the contribution of pilus-1 to virulence. In addition, the discovery of this byphasic phenotype points toward the need to evaluate the expression of the pneumococcal pilus during infection and to understand if *in vivo* conditions will modulate the pilus expression ratio, or if, for still unknown reasons, the coexistence of the two heterogeneous sub-populations is necessary to exploit the pilus virulence potential.

## Materials and Methods

### Bacterial strains and growth conditions

*S. pneumoniae* strains were routinely grown over night (ON) at 37°C in 5% CO_2_ on Tryptic Soy Agar plates (TSA) (Becton Dickinson) supplemented with 10 mg/l colistine, 5 mg/l oxolinic acid, and 5% defibrinated sheep blood. Liquid cultures were carried out statically at 37°C under 5% CO_2_ humidified atmosphere until A_600_  =  0.25 in Todd Hewitt Broth supplemented 0.5% (w/w) yeast extract (THYE) unless otherwise specified (Becton Dickinson). To evaluate pilus-1 expression changes in response to different growth conditions the following experimental settings were used: rich media (THYE, Tryptic Soy Broth, Brain Hearth Infusion broth) or a chemically defined minimal medium [60] supplemented either with MnSO_4_ (1 mM), FeCl_3_ (≥50 mM) or Fetal Bovine Serum (20%); bacteria were also grown until different growth phases (A_600_ ranging from 0.01 to 1.2), at different pH values (5.5, 6.4, 8.4) or in the presence of different O_2_ concentrations.

### Strain collection

A worldwide collection of 1366 strains of *S. pneumoniae* (Figure S4) including both carriage, AOM (acute otitis media) and invasive clinical isolates from different geographical origins, were characterized for serotype (with conventional methods) and sequence type (ST), for the presence of the PI-1 and for PI-1 clade. Multi Locus Sequence Typing (MLST), Clonal Complex (CC) assignment by E-BURST analysis, and PI-1 detection were performed as previously described [28]. The evaluation of pilus expression was performed on a sub-panel of strains randomly selected from among those that resulted PI-1 positive within this collection.

### Genomic DNA extraction and PI-1 sequencing

Genomic DNA extractions were performed from 50 ml of bacterial liquid culture by using the Wizard Genomic DNA purification kit following the manufacturer's instructions (Promega). To obtain PI-1 sequences, oligonucleotides matching on homologous regions inside the islands were designed and used to amplify and sequence the PCR products (Table S1). Sequences were obtained by use of an ABI 3730xl DNA Analyzer and assembled with Vector NTI 10.

### Animal immunizations

Animal treatments were done in compliance with the current law, approved by the internal Animal Ethics Committee (AEC numbers 200601, 200602, 200607 and 200911) and authorized by the Italian Ministry of Health. To generate sera against the specific proteins, purified recombinant proteins were used to immunize CD1 mice (20 µg, three doses administered intra-peritoneally two weeks apart) or New Zealand rabbits of 2.5 kg body weight (100 µg, three doses subcutaneous immunization two weeks apart) (Charles River Laboratory). Two weeks after the third immunization the animals were bled to obtain the sera. A rabbit polysaccharide multivalent antiserum (OMNIserum) was purchased from Staten Serum institute (Copenhagen).

### Flow Cytometry on whole bacteria

Bacteria recovered from liquid cultures were stained with mouse antisera raised against pilus-1 components or surface exposed proteins (final dilution 1∶300). After labelling with a secondary FITC conjugated antibody (Jackson Laboratories, dilution 1∶100), bacteria were fixed with 2% paraformaldehyde. Bacterial staining was analyzed by using a FACS-Calibur cytometer (Becton Dickinson). Sera from mice immunized with PBS (Phosphate Buffered saline) plus adjuvant were used as a negative control. To test the pilus-1 expression in the presence of antibodies directed against RrgB, the bacteria were grown from an A_600_ of 0.01 to 1.2 in THYE supplemented with anti-RrgB rabbit sera at different dilutions (1∶20, 1∶50, 1∶100). The growth was also carried out with rabbit anti-BgaA and with sera derived from animals immunized with adjuvant only (1∶20, 1∶50, 1∶100), used as negative controls. When the desired A_600_ was reached, bacteria were processed for FACS analysis as reported above by using mouse anti-RrgA as primary antibody.

### Immuno-fluorescence staining

Bacteria were harvested form a plate after an ON growth, washed with PBS pH 7.4, fixed with 2% (w/v) paraformaldehyde in PBS and then attached to polylysine-coated cover slips. After washing five times with PBS the slides were blocked for 15 min with PBS 3% BSA (w/v) (Bovine serum albumin) supplemented with 10% normal goat serum (Sigma). Primary and secondary antibodies conjugated with fluorochromes (Invitrogen) were diluted in PBS containing 1% BSA and incubated with the bacterial cells for 30 min at room temperature. Between incubation steps the bacteria were washed thoroughly with PBS. To reduce bleaching of the fluorochromes, the slides were mounted in Pro Long Gold antifade reagent with DAPI (Invitrogen). Images were obtained using a Carl Zeiss LSM 7MP Laser Scanning Microscope.

### Depletion of RrgB positive bacteria

TIGR4 low pilus expressing bacteria were incubated with rabbit anti-RrgB antibodies (1∶400 dilution) and then with goat anti-rabbit IgG biotin conjugated (Abcam, 1∶1000 dilution) antibodies. Labelled bacteria were then incubated with Sepharose magnetic beads coated with streptavidin (Invitrogen) for 1 h at 4°C. Finally, RrgB positive bacteria attached to the Sepharose beads were removed by placing the tubes in a magnetic separation rack and recovering the bacterial suspension (containing RrgB negative bacteria).

### SDS-PAGE and Western Blot analysis

SDS-PAGE analysis was performed on whole bacterial lysates using Nu-PAGE™ 4-12 Bis-Tris or 3-8% Tris-acetate gradient gels (Invitrogen) according to the manufacturer's instructions. Hi-Mark™ pre-stained HMW protein standard (Invitrogen) served as a protein standard. Gels were processed for Western Blot analysis by using standard protocols. Mouse and rabbit antibodies raised against recombinant His-Tag-proteins were used at 1∶3000 and 1∶5000 dilutions, respectively. Secondary goat anti-mouse and anti-rabbit IgG alkaline phosphatase conjugated antibodies (Promega) were used at 1∶5000 and the signal developed by using Western Blue Stabilized Substrate for Alkaline Phosphatase (Promega).

### Colony immuno-blot

Bacteria were diluted on blood-agar plates to obtain isolated colonies. Before plating the bacteria underwent three sonication cycles (30 seconds, power 50% with a SONICS vibra-cell sonicator), to ensure that the colonies were derived from single bacteria. The effectiveness of sonication was checked by inspection of the bacteria under the microscope before and after the treatment. Following ON growth, nitrocellulose membrane discs (Millipore) were gently placed on the plates, removed after 5 min, heat-treated with microwave irradiation (300 W for 2 min) and then processed for Western Blot analysis as described above.

### RNA extraction

RNAprotect bacterial reagent (Qiagen) was added to 2 ml liquid bacterial culture (2∶1) and the mixture vortexed for a few seconds. After 5 min at room temperature the bacterial pellet was recovered by centrifugation (5000 *g*, 10 min), resuspended in 1 ml prewarmed (100°C) SDS solution (SDS 2%, 16 mM EDTA pH 8.00) and incubated at 100°C for 2 min, under vigorous shaking. Prewarmed (65°C) acid phenol (1 ml) was then added to the samples, that were incubated for 5 min at 65°C under shaking, and then extracted twice with 1 ml Phenol/Chloroform/Isoamyl alcohol (25∶24∶1) and once with 1 ml Chloroform/Isoamyl alcohol (24∶1). The aqueous phase containing the RNA was recovered by sample centrifugation and the RNA precipitation was carried out by adding 2.5 volumes of ethanol and 1/10 vol of Na Acetate 3 M (pH 4.5). After 2 h of incubation at −20°C and centrifugation (16000 *g*, 20 min), the RNA pellet was recovered and purified by using RNeasy Mini Kit (Qiagen) according to manufacturer's instructions. Before the final elution, all RNA samples were subjected two times to a DNase I treatment (Qiagen). Total RNA integrity check was performed on agarose gel.

### Microarray design

Gene expression analysis was performed by using a custom made microarray based on dsDNA fragments (200–500 bp), PCR-amplified on TIGR4 genomic DNA. In the design were also included amplicons matching on specific genes selected from additional five *S. pneumoniae* strains (R6, G54, 70585, P1031 and Taiwan-19F 14). Genes were considered non-specific if they had a corresponding homologous gene in TIGR4 with an identity greater than 80% on at least 80% of the gene length. An additional stringency criterion was applied for the gene selection from 70585, P1031 and Taiwan-19F 14 genomes: genes matching with the amplicons already designed on TIGR4, R6 and G54, having an identity of 87% on at least 70 bp of length, and having lengths shorter than 180 bp, were removed. Furthermore, amplicons corresponding to the genes present in two D39 strain plasmids (pDP1 and pSMB1) and in the serotype 2 capsule biosynthesis locus (D39), as well as those corresponding to the PI-1 genes specific for Clade II (Finland 6B-12) and Clade III (Taiwan 23F-15) were added.

Amplification primers were designed by *Primer3* software (v. 1.0b) [61]. Usually, one pair of primers was designed for each gene; in the case of 337 genes in TIGR4 and 19 genes in R6, multiple primer pairs (ranging from 2 to 7) were designed on the same gene. The resulting coverage in TIGR4 is of 2121/2236 (94.6%) predicted open reading frames (25 fragments cover 2 or more genes that are contiguous, extremely similar or paralogous). For the other *S. pneumoniae* strains, the primer pairs designed on specific genes cover the following number of open reading frames: 144 (R6), 22 (G54), 126 (70585), 120 (P1031), 114 (19F Taiwan 14), 5 (D39 pDP1 and pSMB1) 15 (D39 capsule), 6 (PI-1 Clade II and Clade III). The possible resulting coverage was rechecked by sequence homology between the amplicons and the predicted genes in the additional *S. pneumoniae* strains, requiring at least 70 bp of alignment with an identity of at least 87%. The resulting coverage, based on NCBI annotations is: R6 1879/2043 (92%), G54 1911/2047 (93.4%), 70585 1890/2323 (81.4%), P1031 1955/2254 (87.8%), 19F Taiwan 14 1917/2205 (86.9%).

PCR amplifications were performed on the genomic DNAs (prepared as described above), purified using QIAquick-96 PCR purification plates (Qiagen), eluted in ddH2O before to be checked by gel electrophoresis on a 96-well format and finally diluted with DMSO (dimethyl sulfoxide) 50% (vol/vol). All the PCR-amplified fragments were spotted in quadruplicate by using a Microgrid II spotter (Arrayit Corporation) on Type-VII* aluminium-coated mirrored slides (ArrayJet). Two hundred and one spots were spotted with a higher number of replicas (varying from 8 to 32). Negative controls, such as PCR-processed empty buffer (spots indicated as ‘H2O’) and spotter-processed empty buffer (spots indicated as ‘empty’), were also included.

The chip layout was submitted to the EBI ArrayExpress and is available with the identifier A-MEXP-2001.

### Probe labeling and microarray hybridization

For RNA labeling, 1 µg total RNA, prepared as described above, was reverse transcribed for two hours at 42°C using Super Script II Reverse Trascriptase (Invitrogen), random nonamer oligonucleotides (GE Healthcare) and the fluorochromes Cyanine3-dCTP or Cyanine5-dCTP (GE Healthcare). Following the reverse transcription reaction, labeled cDNA was treated with RNAse (RNAse One, Promega and RNAse H, Invitrogen) at 37°C for 30 min and than purified by using the Qiaquick PCR purification kit (Qiagen), according to the manufacturer's instructions. The incorporation efficiency of the Cy3 or Cy5 dCTP was measured by NanoDrop analysis. Following heat denaturation (2 min at 95°C), equal amounts of Cy5- and Cy3-labeled cDNAs were used to hybridize microarray slides (over night incubation at 42°C) in microarray hybridization buffer (GE Healthcare) and 50% formamide. Slides were then washed once for 5 min in SSC solution (150 mM NaCl and 15 mM Sodium Citrate) 0.2% SDS, and twice for 10 min in 0.1x SSC 0.2% SDS. Finally, the slides were dipped 5 times in 0.1X SSC, 2 times in water and then dried with nitrogen. Images were acquired with PowerScanner (Tecan) at 5 and 10 µm resolution and analyzed with Genepix 6.1 (Axon laboratories).

### Microarray data analysis

Data normalization was performed with the application *BASE2* [62] by using a lowess transformation, as implemented in the *R* software environment, by the *loess* function after an intra-slide median centering and a low intensity spot correction (if the average spot intensity was less than one standard deviation of the background signal the intensity spot was corrected to the same value of one standard deviation of the background signal).

Differential gene expression was assessed by grouping all log_2_ ratio values corresponding to each gene within experimental replicas and spot replicas, and comparing them against the zero value by Student's t-test statistics (one tail). Genes having a t-test *p-value* <0.05 were usually accepted as differentially expressed. Type-I error rate was estimated by *q-value* method [63]. A log_2_ ratio threshold filtering was also applied, and genes with a log_2_ ratio >1 or <−1 were accepted and classified as being significantly changed. The threshold was inferred from log_2_ ratio distribution widths (standard deviation between 0.25 and 0.44 and an average of 0.31) observed in each sample.

Hierarchical clustering was applied as implemented by *MeV* software (v. 4.2) [64] using the Euclidean metrics and the average agglomeration method.

Microarray data were submitted to the EBI ArrayExpress and are available with the identifier E-TABM-1154.

Gene expression changes were validated by quantitative real-time PCR (qRT_PCR) analysis. The primers used for qRT-PCR analysis are reported in Table S2. The qRT-PCR reaction was performed in a Light Cycler 480 II (Roche) by using the Light Cycler RNA amplification kit SYBR green I (Roche) according to the manufacturer's instructions. For each gene, duplicate reactions were performed on the RNA samples isolated from separate assays. Analyses were performed with Light Cycler® 480 SW 1.5 (Roche). The relative quantitation method (threshold cycle ΔΔ*CT*) was used to evaluate the quantitative variation in gene expression between the high and low pilus expressing subpopulations for the 5 different strains tested, relative to each gene examined. The *S16* and *GAPDH* (glyceraldehyde 3-phosphate dehydrogenase) amplicons were used as the endogenous control for the normalization of the data.

### Expression of RrgB or RlrA in TIGR4 low pilus expressing bacteria

The *rlrA,* the *rrgB* and the *srtC-2* genes were amplified from chromosomal DNA of TIGR4 strain by PCR by using the primers listed in Table S3. The PCR products were cloned into the complementation plasmid pMU1328 between BamHI and SalI restriction sites [51]. Expression of RlrA, RrgB and SrtC-2 was under the control of the erythromycin constitutive promoter (Pc), which was amplified with the primers listed in Table S3 and cloned immediately upstream *rlrA, rrgB* or *srtC-2* (EcoRI, BamHI). All plasmids were confirmed by sequencing, and then transformed into TIGR4 low pilus expressing bacteria by conventional methods. Transformant selection was performed by supplementing media with erythromycin (1 µg/ml). Bacteria containing the pMU1328 *Pc_rlrA, Pc_rrgB* or *Pc_srtC-2* plasmids were analyzed by PCR. Expression of pili on the bacterial surface was detected by Western blot, FACS and immune-fluorescence analysis of whole cell lysates.

## Supporting Information

Figure S1 **Pilus expression ratio is constant at different growth phases.** Bacteria expressing pilus-1 of clade I (A, TIGR4), clade II (B, 6B Finland 12) or clade III (C, 35B SME15) were grown in THYE at different A_600_ (0.02, 0.1, 0.25, 0.5 and 1) and labeled with clade specific anti-RrgB antibodies (1∶400 dilution), and FITC anti-mouse IgG secondary antibodies (1∶100 dilution). Pilus-1 expression was then analyzed by flow cytometry (FACS-Calibur).
(TIF)

Figure S2 **Microarray expression profile analysis of the high versus the low pilus expressing sub-populations. A)** Hierarchical clustering representation of complete microarray data. Blue bars indicate genes significantly differentially regulated in at least one strain. **B)** Gene expression profiling of the genes differentially regulated in A. Numbers represent the log_2_ ratios. * P<0.05. **C)** Pilus-1 expression repressors reported in the literature are not differentially expressed**.** Gene expression profiling of high versus low pilus expressing sub-populations for strains TIGR4 (Clade I), 19F Taiwan 14 (Clade I), OREP4 (Clade I), 6B Finland 14 (Clade II) and 35B SME 15 (Clade III), by spotted DNA microarray analysis. The data are measures of relative gene expression in *in vitro* growth liquid cultures. Red and green represent high and low experimental high/low pilus expression ratios for the 5 strains tested, respectively (see scale bar). The columns represent arrays of different strains (two hybridizations were performed with independently prepared samples), and the rows represent the genes. Red and green correspond to high and low experimental high/low pilus expression ratios for the 5 strains tested, respectively (see log_2_ ratio scale bar).
(TIF)

Figure S3 **SrtC-2 is expressed and functional in bacteria transformed with** ***pMU1328-Pc-srtC-2*** **.** WB analysis performed using polyclonal mouse antisera against RrgB and SrtC-2 on whole bacterial lysates shows that: SrtC-2 is expressed in the TIGR4L sub-population when TIGR4L is transformed with *pMU1328 Pc_srtC-2;* SrtC-2 expression does not influence RrgB expression; and the over-expression of SrtC-2 in TIGR4Δ*srtC-1-3* expressing RrgB in a monomeric form, restores RrgB polymerization. Samples were loaded as follows: TIGR4 wt (lane 1), TIGR4Δ*srtC-1-3* (lane 2) and TIGR4Δ*srtC-1-3* transformed with *pMU1328-Pc-srtC-2* (lane 3), TIGR4L transformed with *pMU1328* empty vector (lane 4), *pMU1328-Pc-srtC-2* (lane 5), *pMU1328-Pc-rlrA* (lane 6), or *pMU1328-Pc-rrgB* (lane 7).
(TIF)

Figure S4 **Composition of the Novartis** ***S. pneumoniae*** **global collection.** Geographical origin, number of strains and disease outcome are indicated (ID: invasive disease, C: carriage, AOM: acute otitis media).
(TIF)

Table S1 **Oligonucleotides used to amplify and sequence PI-1 islets of clade I, II and III.**
(DOCX)

Table S2 **Oligonucleotides used in qRT-PCR to validate microarray data.**
(DOCX)

Table S3 **Oligonucleotides used to constructs pMU1328 plasmids expressing RrgB or RlrA.** Underlined sequences correspond to the restriction sites used for cloning.
(DOCX)

Text S1 **Generation of a TIGR4** ***srtC1-3*** **deletion mutant.** Detailed description of the method used to generate the pneumococcal mutant.
(DOCX)
