# Supplementary figures and images for: The Streptococcus pneumoniae Pilus-1 Displays a Biphasic Expression Pattern
Source: PLoS One. 2011 Jun 22;6(6):e21269. doi: 10.1371/journal.pone.0021269 (PMC3120856; doi:10.1371/journal.pone.0021269)

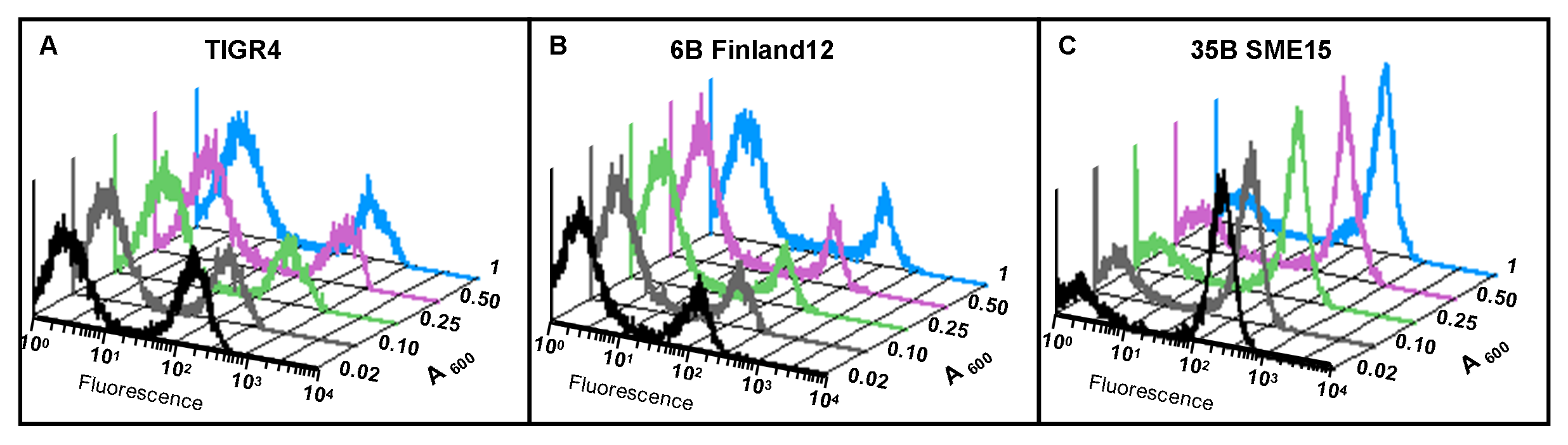

Supplement: Figure S1 — Pilus expression ratio is constant at different growth phases. Bacteria expressing pilus-1 of clade I (A, TIGR4), clade II (B, 6B Finland 12) or clade III (C, 35B SME15) were grown in THYE at different A600 (0.02, 0.1, 0.25, 0.5 and 1) and labeled with clade specific anti-RrgB antibodies (1∶400 dilution), and FITC anti-mouse IgG secondary antibodies (1∶100 dilution). Pilus-1 expression was then analyzed by flow cytometry (FACS-Calibur). (TIF) [file pone.0021269.s001.tif]

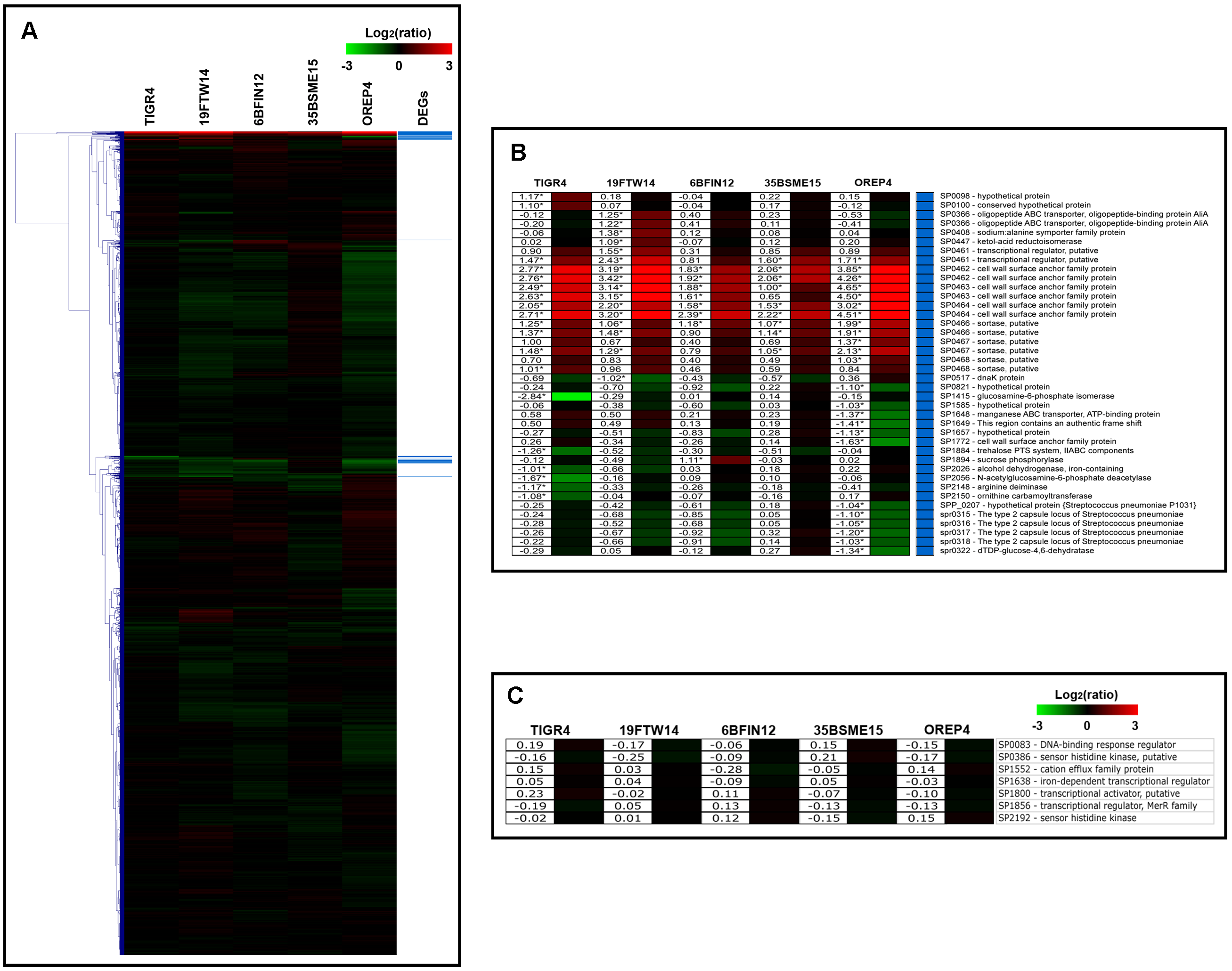

Supplement: Figure S2 — Microarray expression profile analysis of the high versus the low pilus expressing sub-populations. A) Hierarchical clustering representation of complete microarray data. Blue bars indicate genes significantly differentially regulated in at least one strain. B) Gene expression profiling of the genes differentially regulated in A. Numbers represent the log2 ratios. * P<0.05. C) Pilus-1 expression repressors reported in the literature are not differentially expressed. Gene expression profiling of high versus low pilus expressing sub-populations for strains TIGR4 (Clade I), 19F Taiwan 14 (Clade I), OREP4 (Clade I), 6B Finland 14 (Clade II) and 35B SME 15 (Clade III), by spotted DNA microarray analysis. The data are measures of relative gene expression in in vitro growth liquid cultures. Red and green represent high and low experimental high/low pilus expression ratios for the 5 strains tested, respectively (see scale bar). The columns represent arrays of different strains (two hybridizations were performed with independently prepared samples), and the rows represent the genes. Red and green correspond to high and low experimental high/low pilus expression ratios for the 5 strains tested, respectively (see log2 ratio scale bar). (TIF) [file pone.0021269.s002.tif]

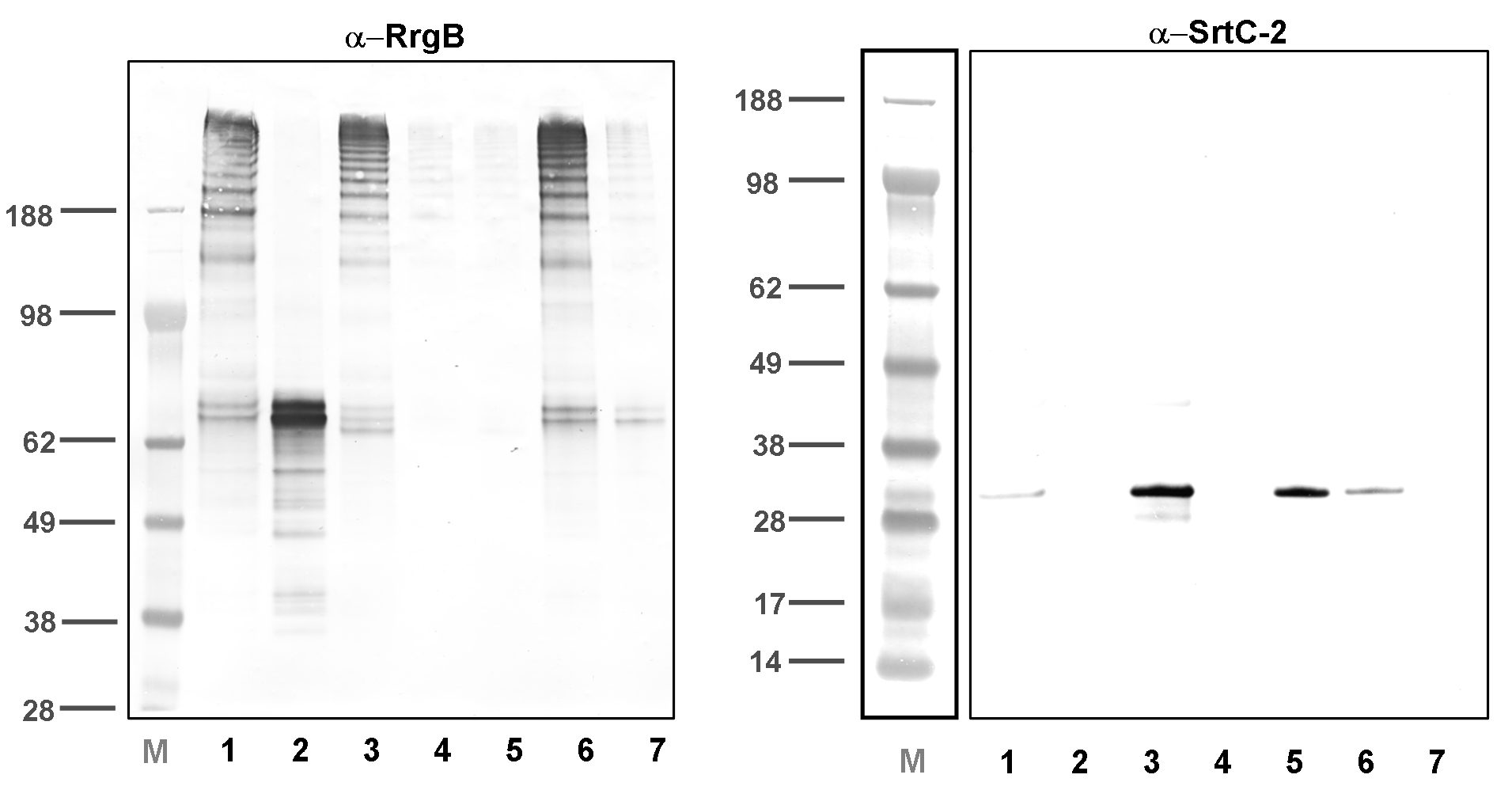

Supplement: Figure S3 — SrtC-2 is expressed and functional in bacteria transformed with pMU1328-Pc-srtC-2 . WB analysis performed using polyclonal mouse antisera against RrgB and SrtC-2 on whole bacterial lysates shows that: SrtC-2 is expressed in the TIGR4L sub-population when TIGR4L is transformed with pMU1328 Pc_srtC-2; SrtC-2 expression does not influence RrgB expression; and the over-expression of SrtC-2 in TIGR4ΔsrtC-1-3 expressing RrgB in a monomeric form, restores RrgB polymerization. Samples were loaded as follows: TIGR4 wt (lane 1), TIGR4ΔsrtC-1-3 (lane 2) and TIGR4ΔsrtC-1-3 transformed with pMU1328-Pc-srtC-2 (lane 3), TIGR4L transformed with pMU1328 empty vector (lane 4), pMU1328-Pc-srtC-2 (lane 5), pMU1328-Pc-rlrA (lane 6), or pMU1328-Pc-rrgB (lane 7). (TIF) [file pone.0021269.s003.tif]

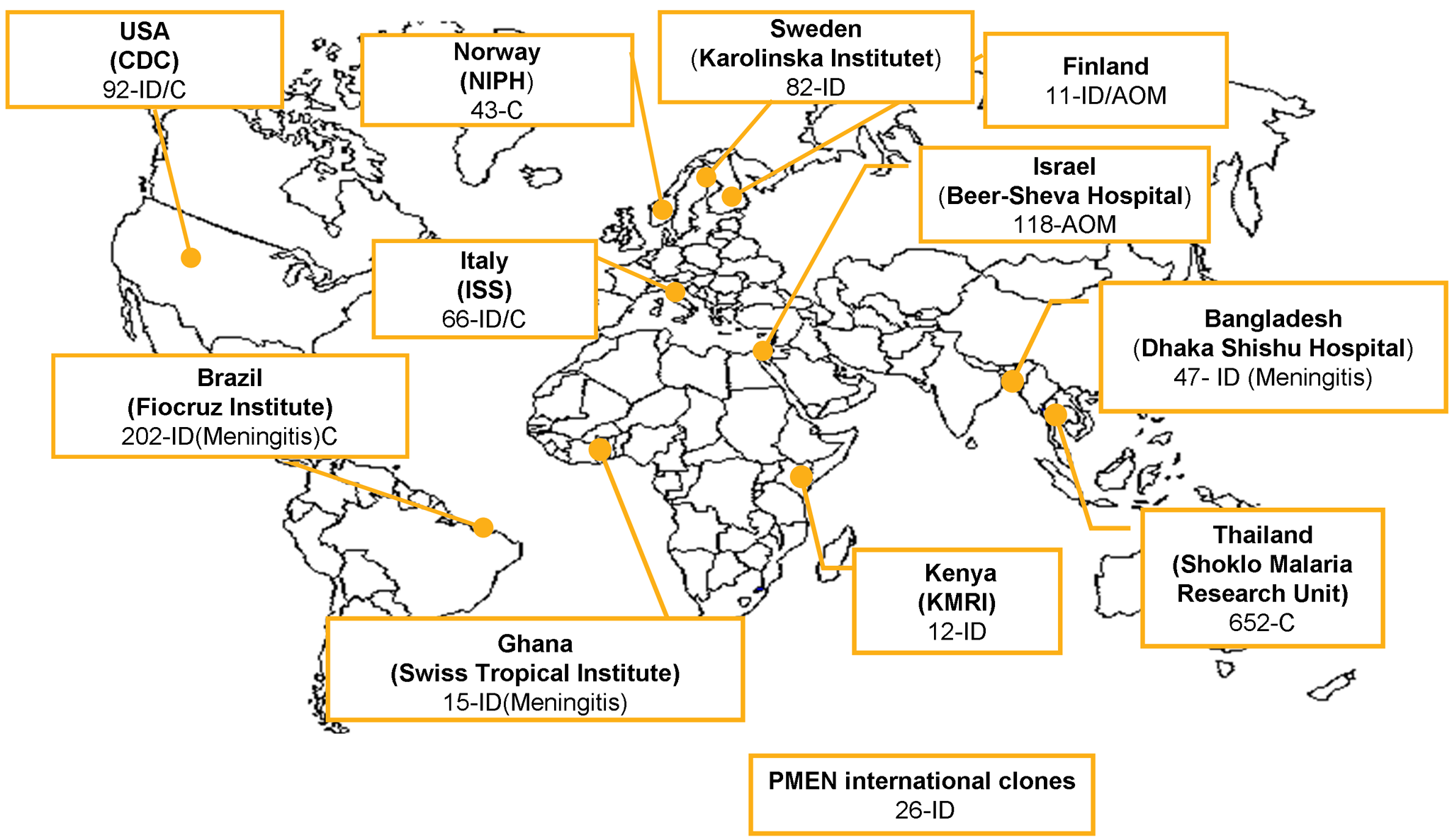

Supplement: Figure S4 — Composition of the Novartis S. pneumoniae global collection. Geographical origin, number of strains and disease outcome are indicated (ID: invasive disease, C: carriage, AOM: acute otitis media). (TIF) [file pone.0021269.s004.tif]
