# Supplementary material for: The Streptococcus pneumoniae Pilus-1 Displays a Biphasic Expression Pattern
Source: PLoS One. 2011 Jun 22;6(6):e21269. doi: 10.1371/journal.pone.0021269 (PMC3120856; doi:10.1371/journal.pone.0021269)
Supplement: Table S1 — Oligonucleotides used to amplify and sequence PI-1 islets of clade I, II and III. (DOCX) [file pone.0021269.s005.docx]

**Table S1 Oligonucleotides used to amplify and sequence PI-1 islets of clade I, II and III.**
