# Supplementary material for: The Streptococcus pneumoniae Pilus-1 Displays a Biphasic Expression Pattern
Source: PLoS One. 2011 Jun 22;6(6):e21269. doi: 10.1371/journal.pone.0021269 (PMC3120856; doi:10.1371/journal.pone.0021269)
Supplement: Table S2 — Oligonucleotides used in qRT-PCR to validate microarray data. (DOCX) [file pone.0021269.s006.docx]

**Table S2 Oligonucleotides used in qRT-PCR to validate microarray data.**
