# Supplementary material for: The Streptococcus pneumoniae Pilus-1 Displays a Biphasic Expression Pattern
Source: PLoS One. 2011 Jun 22;6(6):e21269. doi: 10.1371/journal.pone.0021269 (PMC3120856; doi:10.1371/journal.pone.0021269)
Supplement: Table S3 — Oligonucleotides used to constructs pMU1328 plasmids expressing RrgB or RlrA. Underlined sequences correspond to the restriction sites used for cloning. (DOCX) [file pone.0021269.s007.docx]

**Table S3. Oligonucleotides used to constructs pMU1328 plasmids expressing RrgB or RlrA.** Underlined sequences correspond to the restriction sites used for cloning.
