# Supplementary material for: The Streptococcus pneumoniae Pilus-1 Displays a Biphasic Expression Pattern
Source: PLoS One. 2011 Jun 22;6(6):e21269. doi: 10.1371/journal.pone.0021269 (PMC3120856; doi:10.1371/journal.pone.0021269)
Supplement: Text S1 — Generation of a TIGR4 srtC1-3 deletion mutant. Detailed description of the method used to generate the pneumococcal mutant. (DOCX) [file pone.0021269.s008.docx]

**Generation of a TIGR4 *srtC1-3* deletion mutant**

A TIGR4 ∆*srtC1-3* isogenic mutant was generated by allelic exchange. Fragments of approximately 500 bp upstream and downstream the target gene were amplified by PCR (upstream fragment primers: for AATTGTCGAC TCTGTTAGGAAAAGCGATAAAATG, rev GTTGGCCACTTAGGCCATCATGACCAGTACCAGCATAAACCGGCAA; downstream fragment primers: for CTAGCCGGCATTTAAATTTGCATCGAACCTCTCAATGGTTGTACCGTGG, rev TTTAGCGGCCGCACCAATAAAGAGATTTTAGACAAG) and spliced into a kanamycin resistance cassette (amplified with the primers: for GTCATGATGGCCTAAGTGGCCAACCTGCAGGAACAGTGAATTGGAGTT, rev CGATGCAAATTTAAATGCCGGCTAGTTAGACATCTAAATCTAGGTACTA ) by using overlap extension PCR; the PCR fragments were then cloned into pGEMt (Promega) and transformed in *S. pneumoniae*. To select the bacteria in which the target genes were replaced with the resistance cassette, bacteria were plated on blood-agar plates with kanamycin (500 µg/ml). The presence of the isogenic mutation was confirmed by PCR and Western blot analysis. To obtain a TIGR4 ∆*srtC1-3* complemented mutant, *pMU1328-Pc-srtC-2* was transformed into TIGR4 ∆*srtC1-3* with conventional methods. Transformants selection was performed by supplementing media with kanamycin (500 µg/ml) and erythromycin (1 µg/ml) and complementation confirmed by Western blot analysis.
